# The Impact of the Six Pillars of Lifestyle Medicine on Brain Health

**DOI:** 10.7759/cureus.34605

**Published:** 2023-02-03

**Authors:** Ecler Jaqua, Edna Biddy, Clare Moore, Genise Browne

**Affiliations:** 1 Family Medicine, Loma Linda University Medical Center, Loma Linda, USA; 2 Geriatrics, University of California Irvine, Irvine, USA; 3 Family Medicine, Loma Linda University School of Medicine, Loma Linda, USA

**Keywords:** sleep habits, physical activity, plant-based diet, lifestyle behaviours, alzheimer’s dementia

## Abstract

Dementia is growing exponentially worldwide. Unfortunately, the treatment available does not reverse any type of cognitive impairment. As a result, healthcare professionals are focusing on other evidence-based options, such as lifestyle medicine (LM). Current evidence demonstrates improvement in neurocognitive decline by applying the six pillars of LM, which include plant-based nutrition, physical activity, stress management, avoidance of risky substances, restorative sleep, and social connections.

Plant-based nutrition has a positive impact on cognition by decreasing the risk for Alzheimer's disease (AD) with high adherence to the Mediterranean-Dietary Approach to Systolic Hypertension (DASH) Intervention for Neurodegenerative Delay (MIND). Physical activity also might prevent neurocognitive decline by increasing fibronectin type III domain-containing protein 5 (FNDC5) and Irisin in the hippocampus, which increases energy expenditure and prolongs endurance.

Additionally, higher perceived stress in adulthood and the use of risky substances such as alcohol, nicotine, and opioids are significantly associated with developing mild cognitive impairment and all-cause dementia. Furthermore, there is a positive correlation between poor sleep and social isolation with a rapid progression in cognitive decline. Lifestyle changes have a substantial impact on brain health. Therefore, the focus should always be on prevention as the primary treatment tool.

## Introduction and background

Currently, over 5 million Americans live with Alzheimer's disease (AD), and one in three older adults die with AD or another neurocognitive disorder. In addition, the cost of care in 2020 was $305 billion and is likely to become triple by 2050 [1,2]. Unfortunately, the available treatments do not reverse any cognitive impairment, but in limited cases may help slow the progression of the disease. As a result, many patients and healthcare professionals are looking for other evidence-based treatment options, such as lifestyle medicine [1,2].

Lifestyle medicine (LM) is a medical specialty that primarily uses lifestyle changes to treat chronic diseases. It is an evidence-based practice that helps individuals and their families implement and maintain healthy behaviors impacting the quality of life. Many studies demonstrate cost-effective solutions to prevent and eventually improve neurocognitive decline by applying the six pillars of LM: plant-based nutrition, physical activity, stress management, avoidance of risky substances, restorative sleep, and social connections [2,3]. In addition, the LM pillars target known modifiable risk factors for major neurocognitive impairment, including hypertension, depression, smoking, insulin resistance, diabetes mellitus type 2, hypercholesterolemia, and obesity [1,3]. This article will discuss how the six LM pillars can prevent and improve neurocognitive impairment in older adults.

## Review

Whole food, plant-based nutrition

Numerous studies have suggested and strongly supported the positive impact of a whole foods diet on cognition. The most common diets associated with cognitive protection include the Mediterranean diet, the Dietary Approach to Systolic Hypertension (DASH), and the Mediterranean-DASH diet Intervention for Neurodegenerative Delay (MIND) [4,5]. 

The Mediterranean diet was modeled after those found in countries such as Greece, where the dietary pattern is influenced by the country's association with the Mediterranean Sea [6]. It focuses on the high consumption of fruits, vegetables, legumes, nuts, cereals, and olive oil, moderate consumption of alcohol and dairy, and restricting the consumption of red meat, processed meat, saturated fats, and sweets [6,7]. This combination of food groups is believed to provide essential micronutrients and fibers and decreases the risk of neurodegenerative diseases, such as AD [6-8].

For example, in one systematic review, the randomized controlled trials reported significant improvement in delayed recall, global cognition, and working memory but no significant improvements in attention, episodic memory, immediate recall, processing speed, paired associates, or verbal fluency. Furthermore, in studies such as The North Manhattan Study and the Bordeaux Three-City Study, more remarkable preservation of white matter microstructure, positive changes to white matter hyperintensities, and increased total brain matter were found in participants who adhered to a Mediterranean diet. Some suggested mechanisms for these findings include the diet's impact on neurovascular health and decreased inflammation and oxidative stress levels [7].

The DASH diet also focuses on plant-based foods but limits the intake of short fatty acids, total fat, cholesterol, sweets, and sodium, and was initially developed to prevent and treat hypertension. In multiple longitudinal studies, the DASH diet was associated with improvements in verbal memory but not necessarily with visual memory or executive function. Additionally, with increased diet adherence, participants were found to have less change in global cognition and episodic and semantic memory over time [6]. One randomized controlled trial also demonstrated a significant increase in cognitive function and better average cognition when the DASH diet was coupled with weight management versus the DASH diet alone.

The MIND diet incorporates the Mediterranean and DASH diet components and was developed with the goal of neuroprotection and prevention of dementia [6,9]. It highlights the consumption of plant-based foods with an emphasis on berries and green leafy vegetables while restricting red meats, pastries, sweets, dairy, and fast-fried foods. In addition, an Australian longitudinal study showed a 53% decreased risk for AD with high adherence to the MIND diet and a 35% decrease in patients with moderate compliance [6].

These dietary patterns provide greater ease of application for individuals seeking a dietary model. Still, research on specific nutritional components offers insight into the impact of the micronutrients found in these diets. Notable nutrients mentioned in various studies include vitamin B12, folate, omega-3s, and antioxidants. For example, a longitudinal study in Sweden evaluated the impact of low vitamin B12 and folate on cognition. Participants were twice as likely to develop dementia over three years [8].

In a randomized controlled trial involving participants with mild cognitive impairment, treatment with high doses of folic acid, vitamin B12, and B6 was shown to lower brain atrophy after two years of treatment. Other studies showed improvements in memory, information processing, sensorimotor speed, and improved clinical response to cholinesterase inhibitors in AD patients. Omega-3 fatty acids are another dietary component found in the Mediterranean diet, specifically in fish that have been studied individually. They have been associated with decreased incidence of cognitive decline, reduced amyloid accumulation, increased brain volume, and a decrease in white matter hyperintensities [8]. Lastly, antioxidants such as vitamins A, C, and E found in fruits, vegetables, nuts, and berries have demonstrated improved cognition, decreased risk of cerebrovascular events, decreased cognitive impairment, and prevention of neurologic dysfunction [8,10]. When considered in the context of a whole-food diet, studies on these micronutrients provide practical implications on why these dietary patterns have effectively prevented or slowed cognitive decline.

Physical activity

Physical activity has many benefits to physical, social, and emotional health and has been recommended for health promotion, especially for older adults. Research shows that regular physical activity helps slow down the progression of major neurocognitive disorders such as AD. In addition, physical activity stimulates brain chemicals and neuronal connections that may protect the brain and decrease with aging. Mobility also helps prevent and actually improve some risk factors for major neurocognitive impairment, such as diabetes mellitus type 2, cardiovascular diseases, hyperlipidemia, and metabolic syndrome [11,12]. 

A few studies showed that physical activity might prevent neurocognitive decline by increasing fibronectin type III domain-containing protein 5 (FNDC5) and Irisin in the hippocampus. Irisin, an exercise-induced hormone, is a fragmented product of the FNDC5 that converts white adipose tissues to brown adipose tissue resulting in increased energy expenditure. In addition, irisin prolongs exercise endurance and decreases insulin resistance, improving overall glucose tolerance [12]. As expected, the FNDC5/irisin levels are diminished in both hippocampus and cerebrospinal fluid in individuals with AD [13,14].

The FNDC5/Irisin pathway enters the brain when it is activated by exercise, triggering a cascade that changes neuronal function. Increased FNDC5/Irisin levels may stimulate the hippocampus's brain-derived neurotrophic factor (BDNF), improving synaptic integrity, neuronal cell survival, learning development, and memory. Furthermore, irisin promotes neurogenesis and suppresses amyloid-beta (Aβ) accumulation [13-15].

Although any type of exercise is beneficial for overall health, multiple studies have established that aerobic exercise, compared to strength training and multimodal activities, is more advantageous for neurocognition in patients with mild cognitive impairment (MCI) [1,16]. The exercise intensity was also studied and demonstrated a positive correlation with extraneous physical activity and increased blood flow to the brain, improving executive function, increasing brain size, and decreasing the risk of neurocognitive decline [1,17].

Of all lifestyle changes, physical activity has shown to be a practical approach to improving and decreasing the risk of dementia. 

Stress management 

Another important component when evaluating a patient at risk for dementia is their stress history. In a recent systemic review and meta-analysis of more than one thousand studies, higher perceived stress in adulthood was significantly associated with developing mild cognitive impairment and all-cause dementia [18,19]. Self-reported stress levels seemed to be a reliable metric in these studies and indicated that the patient was aware of the stress in their life. Interventions for this stress, such as behavioral health, would help process the experience of stress. In the same study, patients with more than two significant stressful life events also had higher rates of all-cause dementia [18].

Interestingly, one significantly stressful life event wasn't enough to correlate with dementia. It could be interpreted that resilience to stress decreases with each successive traumatizing event. In general, helping our patients through stress would be helpful not only in the present but also in reducing the severity of dementia in the future.

There has been a significant amount of research regarding the connection between the late onset of AD and stress. AD is a complex disease in which environmental and genetic factors combined lead to severe cognitive decline. However, recent studies have suggested this late-age disease may be predominately caused by stressors earlier in life, such as lack of access to adequate food, housing, and trauma [19]. Another landmark study still examines the relationship between social factors and late-life AD. A prospective study since 1968 of women in Gothenburg evaluated possible risk factors for dementia. Women with significant psychosocial stress and a higher number of stressful events in mid-life had a statistically significant increase in the rate of major neurocognitive impairment three decades later in life [20]. Psychosocial stressors included being a widow, divorced, limited social connections, receiving help from social security, and having a mental illness in a close relative. Events that occurred many years before old age seemed to have damaging effects on the brain and predisposed women to dementia in the future.

Proper history-taking and follow-up of stress-related risk factors would be recommended for at-risk individuals. In addition, nonpharmacologic interventions may prevent and delay further brain damage [19,20].

Avoidance of risky substances

The use of risky substances, including alcohol, nicotine, and prescription drugs such as benzodiazepines, can potentially impact cognition in the elderly. The effects of chronic alcohol use are complex and range from its impact on nutrition to structural changes in the brain. Much is still unknown regarding its effects on the progression or worsening of cognitive impairment in the elderly, but multiple studies have come to similar conclusions [21]. One study published by the Lancet Commission identified alcohol consumption as a modified factor in preventing dementia. A five-year longitudinal study and a systematic review found an association between alcohol use disorder and increased dementia risk (especially the early onset) [22]. However, light to moderate alcohol consumption was actually linked to a decreased risk of dementia [22-24]. The protective factor of alcohol has been recently called into question due to possible socioeconomic class and intelligence confounding factors [23,24]. Additionally, the definition of light and moderate alcohol consumption has been challenging and controversial to quantify [23].

Various studies have proposed multiple mechanisms explaining the pathophysiology of AD in the setting of chronic alcohol use [24]. It has been suggested that chronic alcohol use increases cytokines, toll-like receptor activation, prostaglandins, inducible nitric oxide synthase (iNOS), and microglia activation, leading to neurodegeneration and neuronal loss. Additionally, it is hypothesized that alcohol is involved with the aggregation of Aβ and subsequent neurodegeneration in a complex, self-perpetuating cycle [25]. This cycle requires microglia to act as a protective factor by consuming Aβ aggregates and later a destructive element when overloaded and releasing inflammatory cytokines [24,25]. 

The second notable substance regarding cognition in the elderly is nicotine. When describing the impact of smoking on dementia, it should be noted that studies often focus on former, current, and ever (both former and current) smokers and the risk of AD, all-cause dementia, and vascular dementia [26,27]. A meta-analysis of prospective cohort studies found that the risk of cognitive impairment was significantly increased in current and ever smokers compared to never or former smokers [27]. Another systematic review reported similar results but found greater significance in the relationship between current smoking and, specifically, AD development [26]. Proposed mechanisms for this relationship include the disruption of homeostasis between the generation and reduction of free radicals leading to increased oxidative stress [27]. Another proposed theory focuses on the known cardiovascular effects of smoking and how it possibly impacts the development of dementia on a microvascular scale [26,27].

Lastly, studies examining the impact of benzodiazepines on the elderly population are valuable because of the increased use of these medications among older adults. For example, in a retrospective cohort study examining benzodiazepines and similar drugs, the most significant finding was an increased risk of dementia that was most notable in women and participants taking high doses of short half-lives benzo-related drugs [28]. In another retrospective cohort study, similar results were obtained, with participants having an increased risk of dementia using both short-acting and longer-acting benzodiazepines for more than 28 days in a three-month period [29].

Together, these studies provide insight into the impact of these drugs on a population already at risk for cognitive impairment and decline.

Restorative sleep 

Sleeping enables the repair and clearance needed to correct and prevent neuronal damage and helps with learning and synaptic homeostasis [30]. We invest approximately one-third of our life in sleep; however, not all sleep is considered ideal. First, we need to understand the anatomy of sleep, which is divided into four stages N1, N2, N3 (slow sleep), and Rapid Eye Movement (REM), when dreams are created [31]. Sleep will vary according to the different stages of life. As we age, stages of sleep tend to change. For example, the total sleep time shortens from 14 to 7-9 hours in older adults [30,32]. Most changes in sleep patterns occur before the age of 60 years of age [33]. More than 50% of American adults self-reported low sleep quality, which is more common in women >45 years of age, equalizing at the age of 75 [32,33]. Total sleep duration has been seen as a risk for dementia, the ideal sleep duration for adults being between 7 and 9 hours [33,34] (Table 1). 

**Table 1:** Age-Related Sleep Changes [31,35]

Sleep Stages	Age-Related Hallmark Changes
Daytime napping	Increased
Nighttime awakenings	Increased
Percent rapid eye movement (REM)	Decreased
Sleep efficiency (time asleep over time in bed)	Decreased
Sleep latency (time to fall asleep)	Increased or no change
Stage N1 (transition between awake and sleep)	Increased
Stage N2 (throughout the sleep)	Increased
Stage N3 (slow-wave sleep)	Decreased
Total sleep time	Decreased

Physio-pathologically it has been hypothesized that one of the reasons for the decreased need for sleep is that as we age, we lose neurons, including the ones associated with rest. For this reason, sleep is associated with cognitive decline. Also, it has been seen that as we age, we tend to secrete less melatonin which helps with sleep circadian cycle regulation. Extrinsic factors include retirement, different routines, stressors, caffeine intake, and exercising before bedtime [32,35].

Poor sleep quality and sleep disorders, such as insomnia, movement disorders, and obstructive sleep apnea (OSA), are associated with higher cognitive impairment, diabetes mellitus type 2, cardiovascular diseases, obesity, and depression [33,35,36]. Studies show a positive correlation between sleep and a rapid progression in cognitive decline by Aβ buildup in AD and oxidative stress in vascular dementia [33,36]. 

Targeting sleep quality is a good measure of preventing neurocognitive disorders [35]. Nonpharmacological management, including cognitive behavioral therapy for insomnia (CBT-I) and sleep hygiene education, should be considered first-line therapy since it is superior to pharmacologic treatment [32]. See Table 2 for more information about sleep hygiene. Early screening, diagnosis, and treatment for sleep disorders may help decrease the risk of neurocognitive decline and other medical conditions.

**Table 2:** Sleep Hygiene Education [37,38]

Effective Practices for a Healthy Sleep
Avoid caffeine, alcohol, or large dinner before bedtime
Avoid staying in bed if not asleep after 15-20 minutes
Be consistent—wake up at the same time during the weekdays, weekends, and vacations
Cut back screen time 2 hours before bedtime
Decrease fluid intake before bedtime
Exercise regularly during the day
Limit bright and blue light exposure in the evenings
Make the bedroom a comfortable and quiet place with a cool temperature
Sleep 7-8 hours every night
Stop using electronic devices 30-60 minutes before bedtime
Use the bed only for sleep and sex

Social connections

It is essential to consider social connections when evaluating patients with concerns about cognitive impairment. Cognition, specifically the ability to process higher levels of information and memorize long-term and short-term facts, separates humans from other animals. Development of the brain, which includes social connections, also meant that humans were uniquely suited to develop deeper relationships with other humans. It is hypothesized that the social brain evolved as a defense mechanism. Those living in more complex social groups could also perform more complex computations to help cooperate and compete [39,40].

In recent research, there seems to be a connection between increasing social relationships and preserving brain matter. In a randomized control trial in 250 elderly adults, just one-hour sessions of scheduled group discussion three times a week were shown to have a statistically significant increase in brain volume on magnetic resonance imaging (MRI). It also showed increased performance in neuropsychology testing compared with peers without scheduled interaction [40]. This group created such deep connections that they continued to meet for years after the study ended. In another study with MRI, patients with more complex and extensive social networks had larger volumes of the amygdala [41]. It is presumed that it must be challenging to maintain these relationships and thus requires more meaningful use of the brain. Some studies have gone as far as to create 3D "in-degree" depictions of small communities' social networks. Those patients with the most connections towards the center of these social networks had high grey matter density in the orbitofrontal cortex and dorsomedial complex on MRI [42].

Poor social literacy can also be a warning sign of cognitive impairment. One of the first findings of dementia is social withdrawal, which is associated with worsening social cues and understanding of social situations [43]. Patients with frontotemporal dementia have difficulty interpreting body language and gestures. Consequently, they are insensitive to facial expressions and have trouble understanding sarcasm due to atrophy of the brain [44,45]. Finally, a prospective cohort study of approximately 5,800 older women with low social support showed a two-fold increase in the incidence of all-cause dementia [45]. Individuals with strong social bonds are less likely to develop cognitive impairment, and therefore it is crucial to obtain a comprehensive social history, especially in high-risk older adults.

## Conclusions

Lifestyle changes have a significant impact on brain health. Our review showed that simple interventions in the six pillars of LM might prevent, delay, and improve neurocognitive impairment. This includes preventing and managing risk factors such as diabetes mellitus type 2, hypertension, hyperlipidemia, sleep disorders, illicit drugs, and psychiatric factors.

Therefore, we recommend that all primary care physicians provide education and resources about lifestyle changes to middle age and older adult populations with an increased risk of developing major neurocognitive disorders. The focus should always be on prevention as the primary treatment tool.
